# The role of the specialized team in the operation of continuous renal replacement therapy: a single-center experience

**DOI:** 10.1186/s12882-017-0746-8

**Published:** 2017-11-13

**Authors:** Harin Rhee, Gum Sook Jang, Miyeun Han, In Seong Park, Il Young Kim, Sang Heon Song, Eun Young Seong, Dong Won Lee, Soo Bong Lee, Ihm Soo Kwak

**Affiliations:** 1 Department of Internal Medicine, Pusan National University School of Medicine, Busan, Republic of Korea; 2 Biomedical Research Institute, Pusan National University Hospital, Busan, Republic of Korea; 3 Department of Nursing, Pusan National University Hospital, Busan, Republic of Korea; 4 Division of Nephrology, Pusan National University Hospital, Gudeok-ro179, Seo-gu, Busan, Republic of Korea 602-739

**Keywords:** Acute kidney injury, Continuous renal replacement therapy, Specialized team intervention

## Abstract

**Background:**

The requirement of continuous renal replacement therapy (CRRT) is increasing with the growing incidence of acute kidney injury (AKI). The decision to initiate CRRT is not difficult if an adequate medical history is obtained. However, the handling and maintenance of CRRT constitute a labor-intensive intervention that requires specialized skills. For these reasons, our center organized a specialized CRRT team in March 2013. The aim of this study is to report on the role of a specialized CRRT team and to evaluate the team’s outcome.

**Methods:**

This retrospective single-center study evaluated AKI patients who underwent CRRT in the intensive care unit (ICU) from March 2011 to February 2015. Patients were divided into two groups based on whether they received specialized CRRT team intervention. We collected information on demographic characteristics, laboratory parameters, SOFA score, CRRT initiation time, actual delivered dose and CRRT down-time. In-hospital mortality was defined by medical chart review. Binary logistic regression analysis was used to define factors associated with in-hospital mortality.

**Results:**

A total of 1104 patients were included in this study. The mean patient age was 63.85 ± 14.39 years old, and 62.8% of the patients were male. After the specialized CRRT team intervention, there was a significant reduction in CRRT initiation time (5.30 ± 13.86 vs. 3.60 ± 11.59 days, *p* = 0.027) and CRRT down-time (1.78 ± 2.23 vs. 1.38 ± 2.08 h/day, *p* = 0.002). The rate of in-hospital mortality decreased after the specialized CRRT team intervention (57.5 vs. 49.2%, *p* = 0.007). When the multivariable analysis was adjusted, delayed CRRT initiation (HR 1.054(1.036–1.072), *p* < 0.001) was a significant factor in predicting in-hospital mortality, along with an increased SOFA score, lower serum albumin and prolonged prothrombin time.

**Conclusions:**

Our study shows that specialized CRRT team intervention reduced CRRT initiation time, down-time and in-hospital mortality. This study could serve as a logical basis for implementing specialized CRRT teams hospital-wide.

**Electronic supplementary material:**

The online version of this article (10.1186/s12882-017-0746-8) contains supplementary material, which is available to authorized users.

## Background

Continuous renal replacement therapy (CRRT) is the most commonly selected modality of renal replacement therapy for the dialysis required for acute kidney injury (AKI) patients in the intensive care unit (ICU) [1, 2]. Globally, the requirement of CRRT is increasing with the growing incidence of AKI [3–5]. According to a nationwide study conducted in Denmark [5], the crude incidence of AKI requiring dialysis increased from 143 per million in 2000 to 360 per million in 2012. Among those patients, the use of CRRT substantially increased, from 27% in 2000 to 57.6% in 2012. In a multinational AKI-EPI study [6], 75.2% of the AKI patients requiring ICU-admitted dialysis received CRRT.

The main advantage of CRRT is better hemodynamic tolerance [7, 8] than conventional hemodialysis, which removes accumulated solute and fluid within a short time. With CRRT, physicians can gradually remove the same amount of accumulated solute and fluid spread over a whole day as long as the machine does not stop. However, in the field, CRRT is often stopped for various reasons, such as a clotted filter or a delay in the exchange of the dialysate bag. For these reasons, only 60–70% of the prescribed dose is actually delivered [9], and the mortality rate remains high even with the use of CRRT in AKI patients.

To better manage the utilization process of CRRT and to obtain better outcomes when CRRT is used, some centers operate specialized CRRT teams with physicians and nurses from their respective disciplines, which showed favorable results on clinical outcomes in AKI patients treated with CRRT in the ICU [10, 11]. Our center organized a specialized CRRT team in March 2013. In this study, we aimed to describe the role of a specialized CRRT team and provide an analysis of the team’s outcome.

## Methods

### Patients

This investigation was a retrospective single-center study based on data consecutively collected from the patients who received CRRT in the ICU from March 2011 to February 2015. We included all patients who received CRRT, including the elective case of CRRT after open cardiac surgery. For the purpose of the analysis, we divided the patients into two groups: patients who were treated with CRRT during the period prior to implementation of the specialized CRRT team and patients who were treated with CRRT during the period after implementation of the specialized CRRT team. Approval to perform anonymous analyses of routinely collected clinical data was obtained with a waiver of informed consent from the Pusan National University IRB Committee [1702–031-051].

### Performance of the CRRT team

**Fig. 1:**
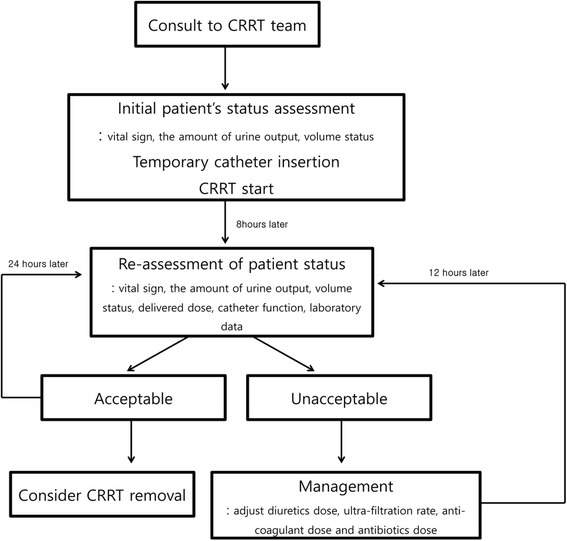
Performance of the specialized CRRT team

The CRRT team was composed of one nephrologist and two specialized nurses who were responsible for the operation and management of the CRRT machine and procedure. The main duty of the CRRT team was to initiate and manage the CRRT, as summarized in Fig. 1. Vascular access was achieved through cannulation of the right internal jugular vein or the femoral vein using an ultrasonography-guided approach. As described in our previous report [12, 13], the CRRT machine was operated using a continuous veno-venous hemodiafiltration (CVVHDF) mode via Prismaflex with an AN 69 ST membrane. Heparin was used as an anticoagulant in most cases, and nafamostat mesylate was used in patients with a propensity toward increased bleeding. In patients with septic AKI, we routinely changed the dialysis membrane every 24 h. The dose of CVVHDF was prescribed as 40 mL/kg/h, equally divided between diffusion and convection. Hemosol was replaced using pre- and post-dilution methods at a proportion of 2:1. The initial blood flow rate was 150 mL/min, and the blood flow rate was increased to 200 mL/min based on patient tolerance. CRRT weaning was considered when the blood pressure had recovered without the assistance of vasopressors or when the amount of urine showed a trend of increasing output [14].

### Data collection and definitions of parameters

Data were collected based on medical chart review. Demographic data, including department residence, baseline biochemical laboratory data and CRRT-associated treatment histories such as the CRRT initiation time, prescribed CRRT dose, actually delivered CRRT dose, CRRT down-time and CRRT operation duration, were collected. The CRRT initiation time was defined as the time from the ICU admission to CRRT initiation [15], and the duration of CRRT operation was defined as the time from the CRRT initiation to the CRRT termination. The CRRT down-time was defined as a period of time when CRRT was not applied between the initiation and end of CRRT [16]. Filter life span was calculated as the actual CRRT operation time (total duration of CRRT operation minus total CRRT down-time) divided by the total number of filters used. We defined premature filter clotting as a filter life span shorter than 12 h. The doses of CRRT that were actually delivered were calculated using the effluent flow rate, with a correction for the percentage of predilution. For the outcome measurement, we evaluated in-hospital mortality and mortality that occurred during the CRRT operation. We defined in-hospital mortality as a death that occurred during the hospital stay and CRRT mortality as a death that occurred during the CRRT operation. We further evaluated renal outcome among CRRT survivors. We defined renal death as a status of dialysis dependence at the time of hospital discharge.

### Statistical analysis

The data were analyzed using SPSS for Windows, version 17.0 (SPSS Inc., Chicago, IL, USA). For continuous variables, the mean ± standard deviation was used to describe normally distributed data, and non-normally distributed data were described using the median. Differences between the two groups were tested using Student’s t-tests for the continuous variables and chi-square tests for categorical variables. Comparisons of the CRRT and in-hospital mortality rate between the two groups were performed using a chi-square test. To identify the role of the specialized team for CRRT and in-hospital mortality, we used binary logistic regression analyses. The choice of which variables to include in the equation was based on the results of the univariable analyses, where each parameter, CRRT and in-hospital mortality, had a demonstrated association (*p* < 0.1). *P*-values less than 0.05 were considered to be statistically significant.

## Results

**Fig. 2:**
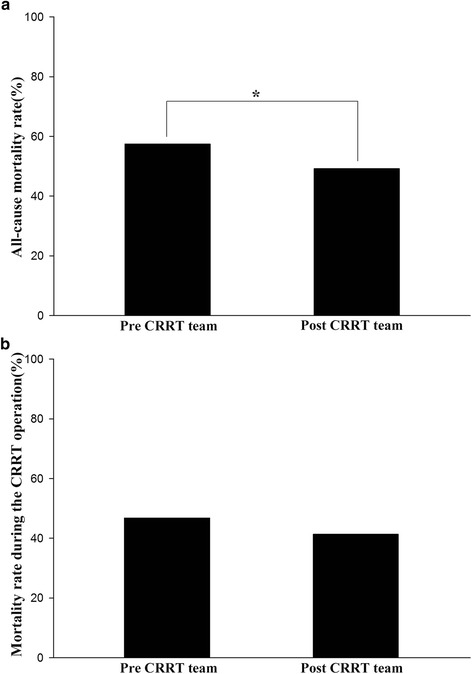
Comparisons of mortality rate between pre- and post-CRRT team intervention; **a**) All-cause mortality rate, **b**) Mortality rate during the CRRT operation. * *P*-value <0.05

A total of 1104 patients were included in this study. Prior to the existence of the CRRT team, CRRT was initiated in 515 patients over a period of two years, with a mean duration of 5.37 ± 5.84 days. During the period when a CRRT team was available, CRRT was initiated in 589 patients over a period of two years, with a mean duration of 5.23 ± 5.66 days. The most common cause for patients to receive CRRT was AKI with septic shock, and the second most common cause was acute pulmonary edema (Table 1). The mean patient age was 63.85 ± 14.39 years, and 62.8% of the patients were males. When we compared patient characteristics according to the CRRT team intervention (Table 2), the mean arterial pressure was slightly higher, the SOFA score was slightly lower, and fewer patients were being mechanically ventilated and being administered vasopressor infusions at the time of CRRT initiation in the period where a CRRT team was available. After CRRT team intervention, there was a significant reduction in both the initiation and down-times for CRRT (Table 3). In the multivariable analyses, specialized CRRT team intervention was independently associated with reduced down-time and initiation time (Additional file 1: Table S1, S2). The frequency of premature filter clotting and the total durations of both ICU and hospital stays were not changed. Due to the changes in the management strategies of CRRT in our clinic, filter life span and actually delivered doses were numerically reduced after the implementation of CRRT team (Table 3). When we performed chi-square tests to evaluate mortality, although the CRRT mortality rate did not change, the in-hospital mortality rate was significantly reduced during the period when a CRRT team was available (Fig. 2). When we analyzed factors associated with the in-hospital mortality rate adjusted for the variable factors described in Table 4, a higher SOFA score, prolonged prothrombin time, delayed initiation of CRRT, longer duration of CRRT operation and lower serum albumin level were associated with increased all-cause in-hospital mortality in these patients. However, the implementation of the CRRT team alone was not a significant factor associated with a reduction in mortality rates in the multivariable analysis (HR 0.858 (0.725–1.016), *p* = 0.076). Similar results were shown when we analyzed factors associated with the CRRT mortality rate (Table 5). We further analyzed renal outcome among survivors. Dialysis dependence rate, serum creatinine level and eGFR and the amount of urine output at the time of discharge were not changed between the two groups (Table 6). Monthly income associated with CRRT operation has increased by 8.8% after implementation of the specialized CRRT team (Additional file 1: Table S3).

**Table 1:** Causes of continuous renal replacement therapy application

Causes	Total (*N* = 1104), %
AKI with septic shock	39.4
AKI with acute brain injury	3.7
AKI without septic shock or acute brain injury	
Acidemia	7.0
Acute pulmonary edema	26.6
Hyperkalemia	2.6
Uremic complications	7.8
Elective CRRT after open cardiac surgery	5.1
Drug intoxication	2.2
Rhabdomyolysis	4.6
Tumorlysis syndrome	1.1

*AKI* acute kidney injury; *CRRT* renal replacement threrapy

**Table 2:** Patient characteristics

	Total (*N* = 1104)	Pre- CRRT intervention (*N* = 515)	Post- CRRT intervention (*N* = 589)	*P*
Dermographic characteristics				
Age, year	63.85 ± 14.39	63.05 ± 14.67	64.56 ± 14.13	0.083
Male, %	62.8	63.9	61.8	0.493
Height, cm	163.59 ± 8.53	163.63 ± 8.10	163.56 ± 8.91	0.884
Weight.kg	61.85 ± 12.07	61.65 ± 12.23	62.03 ± 11.65	0.602
BMI.kg/m^2^	23.07 ± 3.97	22.94 ± 3.82	23.18 ± 4.09	0.320
Surgical ICU, %	19.3	17.5	20.9	0.169
Medical ICU, %	80.7	82.5	79.1	0.169
Disease status				
MAP, mmHg	79.45 ± 17.73	78.01 ± 18.49	80.72 ± 16.95	0.012
SOFA score	10.59 ± 3.98	11.03 ± 4.04	10.21 ± 3.90	0.001
Vasopressor use, %	63.5	70.5	57.4	<0.001
Ventilator use, %	61.3	68.0	55.5	<0.001
Laboratory findings				
WBC, 10E3/uL	14.35 ± 10.82	13.78 ± 9.90	14.84 ± 11.55	0.103
Hb, g/dL	10.35 ± 2.40	10.34 ± 2.41	10.36 ± 2.39	0.884
Hct, %	30.71 ± 7.54	30.93 ± 8.02	30.52 ± 7.09	0.373
Platete, 10E3/uL	143.52 ± 102.86	131.03 ± 93.10	154.4 ± 109.60	<0.001
Total protein, g/dL	5.47 ± 1.09	5.36 ± 1.11	5.56 ± 1.08	0.004
Albumin, g/dL	2.95 ± 0.69	2.89 ± 0.69	3.02 ± 0.67	0.001
BUN, mg/dL	53.91 ± 31.46	55.55 ± 33.51	52.48 ± 29.51	0.106
Cr, mg/dL	3.59 ± 2.79	3.57 ± 2.86	3.61 ± 2.73	0.816
Na, mmol/L	138.06 ± 8.91	138.16 ± 10.25	137.97 ± 7.56	0.727
K, mmol/L	4.51 ± 1.10	4.53 ± 1.12	4.50 ± 1.08	0.696
TCO2, mmol/L	16.74 ± 6.13	15.84 ± 5.92	17.52 ± 6.21	<0.001
PT, INR	1.65 ± 0.97	1.66 ± 1.05	1.64 ± 0.88	0.688

*CRRT* continuous renal replacement therapy; *BMI* body mass index; *ICU* intensive care unit; *MAP* mean arterial pressure; *SOFA* sequential organ failure assessment; *Hb* hemoglobin; *BUN* blood urea nitrogen; *Cr* creatinine; *tCO2* total carbon dioxide; *PT* prothrombin time

**Table 3:** Comparisons of CRRT treatment pattern and patient outcomes between before and after the implementation of CRRT team

	Pre- CRRT intervention (*N* = 515)	Post- CRRT intervention (*N* = 589)	*P*-value
CRRT treatement pattern			
Initiation time, day	5.30 ± 13.86	3.60 ± 11.59	0.027
Prescribed dose, mL/hr	Fixed dose 2000/3000(sepsis)	40 ml/kg	NA
Actual dose, mL/kg/hr	35.31 ± 9.75	33.99 ± 7.51	0.011
Number of used filter, n	4.03 ± 8.86	4.55 ± 4.67	0.225
Filter life span, hrs	24.04 ± 18.16	19.59 ± 12.50	<0.001
Premature filter clotting, %	28.3	27.0	0.628
Total CRRT down time, hr	13.06 ± 26.67	8.49 ± 13.61	<0.001
Down time per day, hr	1.78 ± 2.23	1.38 ± 2.06	0.002
CRRT duration, day	5.37 ± 5.84	5.23 ± 5.66	0.696
Patient outcomes			
Total ICU stay, day	16.60 ± 22.15	15.67 ± 39.85	0.625
Total hospital stay, day	31.00 ± 43.67	32.67 ± 51.20	0.558
All-cause mortality rate, %	57.5	49.2	0.007
CRRT mortality rate, %	46.8	41.3	0.068

*ICU* intensive care unit, *CRRT* continuous renal replacement therapy

**Table 4:** Factors associated with all-cause mortality

	Univariated analysis			Mulivariated analysis		
Variables	HR	95% CI	*P*	HR	95% CI	*P*
Age, yr	1.005	0.977–1.014	0.230			
Male, %	1.034	0.840–1.321	0.788			
BMI, kg/m^2^	0.981	0.952–1.011	0.204			
CRRT team intervention	0.718	0.566–0.910	0.006			
SOFA score	1.327	1.275–1.381	<0.001	1.271	1.202–1.316	<0.001
CRRT initiation time, day	1.019	1.007–1.030	0.001	1.054	1.036–1.072	<0.001
Actually delivered dose, mL/kg/hr	1.021	1.007–1.036	0.003			
Number of filter used	0.957	0.931–0.984	0.002			
Total CRRT operation duration, day	0.999	0.998–1.000	0.088	1.056	1.016–1.098	0.006
Total protein, g/dL	0.643	0.571–0.725	<0.001			
Albumin, g/dL	0.418	0.344–0.507	<0.001	0.604	0.441–0.829	0.002
BUN, mg/dL	0.998	0.994–1.001	0.216			
Cr, mg/dL	0.882	0.841–0.926	<0.001			
Na, mmol/L	1.014	1.000–1.028	0.054			
TCO2, mmol/L	0.972	0.953–0.991	0.004			
PT, INR	2.304	1.814–2.982	<0.001	1.373	1.107–1.703	0.004

Adjusted factors: Age, sex, CRRT team intervention, CRRT initiation time, total CRRT operation duration, number of filter used, actually delivered dose, SOFA score, total protein, serum albumin, serum sodium level and prothrombin time

*Abbreviations*: *BMI* body mass index; *CRRT* continuous renal replacement therapy; *SOFA* sequential organ failure score; *Cr* creatinine; *Na* sodium; *TCO2* total carbon dioxide; *PT* prothrombin time

**Table 5:** Factors associated with CRRT mortality

	Univariated analysis			Mulivariated analysis		
Variables	HR	95% CI	*P*	HR	95% CI	*P*
Age, yr	1.003	0.995–1.012	0.458			
Male, %	1.035	0.809–1.324	0.784			
BMI, kg/m^2^	0.989	0.960–1.020	0.491			
CRRT team intervention	0.798	0.629–1.014	0.064			
SOFA score	1.326	1.274–1.380	<0.001	1.255	1.198–1.314	<0.001
CRRT initiation time, day	1.008	0.998–1.018	0.099	1.057	1.034–1.080	<0.001
Actually delivered dose, mL/kg/hr	1.020	1.006–1.035	0.004			
CRRT down time per day, hr	0.883	0.827–0.942	<0.001			
Number of filter used	0.942	0.913–0.972	<0.001			
Total CRRT operation duration, day	0.968	0.945–0.990	0.006			
Total protein, g/dL	0.625	0.553–0.706	<0.001	0.817	0.667–1.000	0.505
Albumin, g/dL	0.440	0.362–0.534	<0.001	0.708	0.516–0.971	0.032
BUN, mg/dL	0.996	0.992–1.000	0.044			
Cr, mg/dL	0.892	0.848–0.938	<0.001			
Na, mmol/L	1.014	0.999–1.028	0.064			
TCO2, mmol/L	0.970	0.951–0.989	0.003			
PT, INR	2.088	1.693–2.576	<0.001	1.328	1.092–1.616	0.005

Adjusted factors: Age, sex, CRRT team intervention, CRRT initiation time, CRRT duration, number of filter used, actually delivered dose, total protein, serum albumin, serum sodium level, prothrombin time and SOFA score

*Abbreviations*: *BMI* body mass index; *CRRT* continuous renal replacement therapy; *SOFA* sequential organ failure score; *Cr* creatinine; *Na* sodium; *TCO2* total carbon dioxide; *PT* prothrombin time

**Table 6:** Renal outcomes among survivors

	Pre- CRRT intervention (*N* = 217)	Post- CRRT intervention (*N* = 296)	*P*-value
Renal death among CRRT survivors. %	22.9	29.4	0.107
Serum cr. at discharge, mg/dL	2.45 ± 2.12	2.37 ± 2.2.24	0.281
eGFR at discharge, mL/min/BSA	50.47 ± 41.03	48.73 ± 45.81	0.651
Urine output at discharge, L/day	1.28 ± 0.99	1.35 ± 1.11	0.480

*CRRT* continuous renal replacement therapy; *eGFR* estimated glomerular filtration rate

## Discussion

In this study, we evaluated the performance of a CRRT team and factors associated with all-cause and CRRT mortality rates in our clinic. After implementation of the CRRT team, both the initiation and down-times for CRRT were reduced. Similar to previous reports, delayed CRRT initiation [17–19], lower serum albumin [13, 20], prolonged prothrombin time [13] and higher SOFA scores [13, 21] were associated with higher all-cause in-hospital and CRRT mortality rates. Even though the implementation of the CRRT team alone was not a statistically significant factor in predicting in-hospital survival, the all-cause mortality rate was significantly reduced after the CRRT team intervention. Several factors, including the prompt initiation of CRRT, might indirectly affect this favorable outcome after implementation of the CRRT team.

The largest change after the specialized CRRT team in our clinic was the earlier initiation of CRRT. After implementation of the specialized CRRT team, CRRT was initiated 1.7 days faster, and the numeric values of the parameters associated with disease severity at the time of CRRT initiation were significantly reduced: SOFA scores and mean arterial pressures decreased, and the frequency of vasopressor and ventilator use at the time of CRRT initiation decreased. All of these changes indicated that the earlier start time of CRRT after implementation of the specialized CRRT team was beneficial in reducing both in-hospital and CRRT mortality rates. However, the possible beneficial effect of the preemptive initiation of CRRT should be differentiated [17–19]. In previous studies that evaluated the optimal timing for CRRT initiation, early CRRT initiation was defined as the initiation of CRRT in the absence of conventional RRT indications such as acute pulmonary edema, uncontrollable metabolic acidosis or uncontrollable hyperkalemia. In addition, delayed CRRT initiation was defined as the commencement of CRRT after the onset of life-threatening complications [17–19, 22]. In our clinic, CRRT was initiated only when at least one or more of the conventional indications were present, even after implementation of the specialized CRRT team. Although the earlier initiation of CRRT was beneficial in reducing both in-hospital and CRRT mortality rates in this study, the findings do not support the beneficial effect of preemptive CRRT initiation. Instead, the prompt commencement of CRRT in response to the occurrence of life-threatening events appeared to be beneficial in reducing in-hospital and CRRT mortality rates.

The filter span is commonly used as a parameter for quality control, and a longer lifespan is regarded as a marker for good quality control because premature filter clotting is a major problem, increasing blood loss and decreasing actual CRRT delivery in the daily practice of CRRT [23]. It is expected that the filter lifespan would be increased after the implementation of the CRRT team; however, it was decreased in our study because after implementation of the CRRT team, we routinely changed the filter every 24-h for septic patients. In fact, the premature filter clotting rate was not increased after implementation of CRRT team. We routinely changed the filter because the filter itself can remove mid to large molecular weighted solutes by adsorption, which is limited by saturation of the membrane binding sites that can occur within a few hours [24]. Inflammatory mediators such as interleukin 6, interleukin 8 and tumor necrosis factor can be removed by adsorption, according to the molecular weight and degree of plasma protein binding [24]. We believe that by changing the filter, we can increase mid to large molecular clearance, which might have a role in the survival advantage in patients with sepsis or systemic inflammatory response syndrome. Even though we could not prove the direct contribution of routine membrane change to patient survival in this study, our results imply its possible benefit. Further study is needed to discover the optimal filter life span in the daily practice of CRRT.

Before implementation of the CRRT team, the initiation and operation of CRRT were the duties of the attending physician. The CRRT team reduced this workload and helped the physicians focus only on their own job, which was treating patients. Even though we did not measure the reduction in the attending physicians’ workload as a factor in our analysis, it might have played a role in reducing patients’ all-cause mortality rate during the period after the CRRT team was implemented.

The CRRT prescription and operation patterns changed. Before implementation of the CRRT team, a CRRT prescription was fixed at 2000 mL/h or 3000 mL/h (in cases of severe sepsis), regardless of the patient’s body weight. After implementation of the specialized CRRT team, we prescribed a CRRT dose adjusted to the patient’s body weight. To reach a target dose delivery of 20–25 mL/kg/h, following the recommendation made by KDIGO, we prescribed a CRRT dose of 40 mL/kg/h. After implementation of the CRRT team, approximately 85% of the prescribed doses were delivered, which is substantially larger than previously reported studies [9]. The dose that was actually delivered was 35.31 ± 9.75 mL/kg/h during the period prior to implementation of the specialized CRRT team and 33.99 ± 7.51 mL/kg/h during the period after implementation of the specialized CRRT team. With the results of both the ATN [25] and RENAL [26] studies, it is now clear that doses higher than the recommended 20–25 mL/kg/h are not beneficial for patient outcomes. By changing the prescription pattern, we reduced the unnecessary consumption of dialysate and replacement solution, but we clearly needed to do more to reduce the prescription dose. Because approximately 85% of the prescription doses were actually delivered, to reach a target dose, a prescription dose of 25–30 mL/kg/h would be enough in our clinic. Further internal validation studies are needed to examine this issue.

Our study has several limitations. First, we did not collect data regarding patient comorbidities. In our clinic, the mean patient age was 63.85 years, and approximately 56% of the patients were older than 65 years old. Comorbidities such as diabetes, congestive heart failure, cirrhosis of the liver, stroke or malignancy are important factors associated with mortality in elderly AKI patients who require CRRT [13]. However, these baseline characteristics are less likely to change during the study period; thus, there might be a less significant impact in the assessment of the performance of the CRRT team.

Second, we did not exclude patients whose treatment was performed using Prisma, which is an older model of the Prismaflex CRRT machine. We used Prisma until mid-2011. Predilution is impossible with the Prisma. The predilution method is known to have an advantage over the post-dilution method in reducing filter clots and allowing for a longer life-span of the filter, which results in the reduction of CRRT down-time [27]. Thus, this method might have played a role in reducing CRRT down-times during the period after implementation of the specialized CRRT team, regardless of CRRT team intervention. However, the Prisma model was used in only a small fraction of patients in a short period and thus would likely have little influence on our results.

Third, we did not fully compare a renal outcome between two periods. It is an important issue in the operation of CRRT, however, we didn’t analyze factors associated with renal outcome because we could not obtain essential parameters determining renal outcome such as patient’s initial volume status, net ultra filtration rate during CRRT operation and the amount of urine output, due to the retrospective manner of this study. Further prospective study is needed to define this issue.

Despite these limitations, a relatively large number of patients were included in this study, and the wide array of parameters associated with CRRT operation used in this study strengthens the results.

## Conclusions

Our study shows that a specialized CRRT team reduced the initiation time and down-times of CRRT and showed a lower in-hospital mortality rate. This study could serve as a logical basis for implementing specialized CRRT teams hospital-wide.

## Additional file

Additional file 1: Table S1. Multivariable linear regression analysis: Factors correlated with CRRT donw time per day. **Table S2.** Multivariable linear regression analysis: Factors correlated with CRRT initiation time. **Table S3**. Comparisons of financial outcomes between before and after SCT. (DOCX 15 kb)

## Abbreviations

AKI: acute kidney injury
CRRT: continuous renal replacement therapy
CVVHDF: continuous veno-venous hemodiafiltration
ICU: intensive care unit
SOFA: sequential organ failure assessment
