# Supplementary material for: The role of the specialized team in the operation of continuous renal replacement therapy: a single-center experience
Source: BMC Nephrol. 2017 Nov 13;18:332. doi: 10.1186/s12882-017-0746-8 (PMC5683314; doi:10.1186/s12882-017-0746-8)
Supplement: Additional file 1: Table S1. — Multivariable linear regression analysis: Factors correlated with CRRT donw time per day. Table S2. Multivariable linear regression analysis: Factors correlated with CRRT initiation time. Table S3. Comparisons of financial outcomes between before and after SCT. (DOCX 15 kb) [file 12882_2017_746_MOESM1_ESM.docx]

Table S1. Multivariable linear regression analysis: Factors correlated with CRRT donw time per day

|  | b(SE) | β | P-value |
| --- | --- | --- | --- |
| Specialized CRRT team intervention | -0.065(0.018) | -0.105 | <0.011 |
| SOFA score | -0.65(0.018) | -0.120 | <0.011 |
| PT, INR | -0.167(0.068) | -0.075 | 0.014 |

Abbreviations: CRRT, continuous renal replacement therapy; SOFA, sequential organ failure assessment; PT, prothrombin time, Adjusted factors: Age, sex, CRRT team intervention, CRRT initiation time, SOFA score, PT

Table S2. Multivariable linear regression analysis: Factors correlated with CRRT initiation time

|  | b(SE) | β | P-value |
| --- | --- | --- | --- |
| Age, yr | -0.052(0.026) | -0.058 | 0.049 |
| Specialized CRRT team intervention | -1.551(0.765) | -0.061 | 0.043 |
| SOFA score | 0.366(0.101) | 0.115 | <0.001 |
| TCO2, mmol/L | 0.250(0.062) | 0.120 | <0.001 |
| Serum albumin,g/dL | -1.398(0.582) | -0.075 | 0.016 |

Abbreviations: CRRT, continuous renal replacement therapy; SOFA, sequential organ failure assessment; TCO2 total carbon dioxide, Adjusted factors: Age, sex, CRRT team intervention, SOFA score, TCO2 and serum albumin level.

Table S3. Comparisons of financial outcomes between before and after SCT

|  | Before SCT | After SCT | P-value |
| --- | --- | --- | --- |
| Total income, 1000 KRW/month | 67,102.08±1,579.39 | 72,391.83±1,711.16 | <0.001 |
| Total income, constant USD 2015/month | 61,616.50±2,478.26 | 67,076.43±2,309.09 | <0.001 |
| Total cutback amount, 1000 KRW/month | 367.25±140.66 | 294.99±163.05 | 0.107 |
| Total cutback amount, constant USD*/month | 337.09±130.28 | 271.74±148.79 | 0.112 |
| Cutback KRW, constant USD amount rate,% | 0.55±0.21 | 0.41±0.22 | 0.033 |

Abbreviations; SCT, specialized continuous renal replacement therapy team; KRW, Korean Won; USD, United States dollar *We calculated constant USD through consumer price index using Bureau of Labor Statistics(http://www.bls.gov/data/).
